# Camp Douglas

**Published:** 1862-10

**Authors:** 


### CAMP DOUGLAS.

One of the first questions asked a patient, "How long have you been sick?" generally gives the answer, "ever since I was taken prisoner." "What is the matter?" "Diarrhœa." There is nothing which so effectually takes the self-satisfaction out of a physician as treating chronic diarrhœa. Add to its usual obstinacy the necessary lack of suitable food, and the stolid indifference of men accustomed to sufferings, and our diarrhœas become the rightful recipients of their usual appellation, the camp curse. The prevalence of diarrhœas among the rebel prisoners is absolutely astonishing. In one of my wards, I one day found that nineteen out of twenty then in the ward had diarrhœa. I have never tested it before or since, but presume it would vary but little among the sick in the hospitals. As to its treatment, my own experience gives me little to offer in the chronic cases, while the acute ones yield readily to opiates combined with astringents. Occasionally I have used mercurials with very marked good effect, and also in others quinine has been producive of excellent results. I have used the following in some of my cases with much satisfaction: ℞—Pulv. Opi, ℈j; Pulv. Alum, ʒij. M—Make 20 powders; give one every two or four hours until bowels check.

In the chronic cases, there seemed but little encouragement to close the portals of their exit. No wonder Surgeons kept from active field service, clamor to quit the business, for I think if anything could effectually dry up the last spark of a man's ambition, hope and resolution, it would be to dole out daily remedies for chronic diarrhœa. The incessant "no better" sounds mockery to your science. The long list of specifics fade out quietly before your trials, but like few reliable institutions, they still run themselves. I have tried nothing that I could add to, or place in the specific column. I think if I ventured to believe anything had done any good, it was the formula given above. In some cases, I combined Tinct. of Cantharides with my ordinary diarrhœa formula, and it seemed to do good for a while, but soon fell into the useless brigade.

I used Iron to no purpose. In fact I ransacked the list, with as little satisfaction as tho' I had tried Elecumfundletop. It always sounds impious to me for a man to speak of toying familiarly with this disease. He is aping God.

The degree of emaciation is incredible. In many it would seem to be no difficult matter to teach osteology, and they turn the skeleton for you.

In most of the cases, towards their termination, ulceration of the lower section of the cornea takes place, and it has always in this as well as other diseases, preluded a fatal termination. It seems to be the point in the nutrient barometer, to which, if nature sink, there is no remedy.

These cases, notwithstanding the irritability of the bowels, must not be deprived of a liberal supply of hearty food.

In many cases, articles which would seem entirely inconsistent with the disease, were tolerated with wonderful comfort—such as meats, pickles, vegetables, etc.

The denial of a substantial and free supply of food, I am confident, is injurious in the confined cases. Many cases were post mortemed without eliciting any peculiarity upon which a basis of treatment could be founded.

We have had a class of cases which are new to me, and may be to some of the readers of the JOURNAL.

The first I would see of them, they would be brought into the wards in collapse. Skin cold, shrivelled and blue, difficulty of breathing, great restlessness, the patient tossing himself incessantly from side to side of the bed, almost or entirely pulseless, and every appearance of impending dissolution. Upon inquiry, the patient had been slightly indisposed for a few days, in different ways, probably diarrhœa or chills, and in two cases no such precursors existed—in these they followed drinking copious draughts of ice water. The patient from this was seized with a sense of prostration, and soon the symptoms of collapse as I have enumerated supervened. The first case from its being connected with pretty free diarrhœa and vomiting, I construed into cholera, and used free doses of calomel, camphor, capsicum and opium, and applied mustard almost all over the body, giving whisky freely internally, and saved my patient.

The next cases rather staggered this belief in being unconnected with diarrhœa, and spinal congestion and cerebro-spinal meningitis loomed up. Accordingly, I dry-cupped the spine freely, applied mustard to it and also to the extremities, gave extract of ginger internally, and saved my patient. The balance I have treated in the same way, and saved all of them, some six in number. One of these acted very strangely. He was brought in the ward with seemingly little the matter, his first attack having been, the day before, from drinking freely of ice water—he having fallen senseless out of the door where he was sitting. He was treated in the barracks, and sent to the hospital next day with nothing tangible for trial.

He soon began going about the wards, and during the temporary absence of the nurse, got up and went to a bucket of ice water, and drank his fill. He went back to his bed, and had scarcely got to it when he fell senseless across it. Soon all evidences of life disappeared, and all supposed him dead, and preparations were being made to carry him out, when some one looking at him discovered a slight throbbing of the carotids, when restoratives, mustard, etc., were applied, and he reacted after a few hours, but did not speak until the following day. From this succeeded a stupidity and typhoid dullness with languid circulation, lasting about a week, from which he slowly recovered. When I saw him the day after his second attack, I blistered his head freely, giving him but little medicine.

Where the exact seat of the disease was, I confess I do not feel free in deciding, but am well satisfied of the nervous seat; but whether of the spinal cord and its membranes, or the organic system, and brought on by the shock in the two cases, by cold applied to the stomach, is to me a mystery. Its malarious origin may be doubted, in the fact of there being no periodicity or tendency to return. Low living could not be assigned as the essential cause, for one of the cases was a cook, who lived well and on the best. Some of them recovered rapidly, whilst others gained very slowly. In these cases vitality was at a very low ebb, for the mustard applied to them always blistered, and in some cases gave rise to very tedious and troublesome ulcers.

Another class of cases which were new to me were parotid abscesses. They usually occurred in patients who had been affected with some active form of disease, occurring upon its subsidence. The patient complained of a deep seated and excruciating pain in the parotid region, with very little swelling for several days; at last the parotid gland seemed almost lifted from its bed, and the distension would be very great, and at the end of a week or more the abscess broke into the ear and discharged freely of a very thick but otherwise natural pus. One I opened externally, but no pus was discharged for many days; at last, however, it discharged externally, but not until it had broken into the ear. The surface in this case sloughed around the opening. I cauterized it freely and it seemed to be doing well, but he sank and died. Of four or five cases I remember, but a single one recovered, notwithstanding the most vigorous, sustaining treatment, including tonics, stimulants, broths, etc. It evidently did not cause death by the profusion of the discharge, for, with one exception, it was not very great, but only marked a foregone degree of debility. I regret I had no opportunity of ascertaining the exact point of suppuration, or the reason of its *exitus* in the external ear.

Jaundice has occurred quite frequently, but there has seemed to be no treatment that affected it, to any marked extent, and my cases have done well with no treatment.

Many cases, from their condition of inanition, I am sure labor under softening of the brain and spinal cord. The patients seem affected with few tangible symptoms, except a low or partial delirium, when their attention is arrested conversing sensibly, pulse natural, or perhaps weaker or more irritable; no fever, or thirst; tongue natural, except pale; an indifferent appetite; bowels natural or slightly looser; skin pale or dusky bronze; and in this condition they would lie for from three to four weeks and gradually sink. Many cases in my care, and also in other wards, died very suddenly and unexpectedly; which may be accounted for by these conditions: Patients who were suffering under no violent forms of disease and whose cases gave no anxiety, from the gravity of the symptoms, would surprise by their sudden demise.

Pneumonia gave me new confidence in Tart of Antimony. We are very apt to allow our fears to get the better of our judgment, and in assuming charge of as frail, forlorn and vitiated a set of bodies as belong to the camp rebels antiphlogistics took to themselves wings. But, from a careful venture, soon rested securely with Tartar Emetic as my sheet anchor in Pneumonia. Occasionally we were out of medicines, and in one of these emergencies, I ventured on the following useful, if not elegant prescription: ℞. Vin. Antim ℥jss; Tr. Opii ℥ss. M. Give thirty drops every two hours.

This venture, brought out by the necessity, formed the basis of my practice, and I have lost but two cases since its use. I sometimes vary it by substituting Morphia for the Laudanum.* We were but poorly gratified when we found Dysentery making its appearance. It seemed with our patients, as a class, it must make sad havoc. But I am gratified to say that I have been very agreeably disappointed. Many of these cases were very severe, and there seemed but little hope of relief, but nearly all the cases recovered—a very small per cent dying. I give to the following formula the credit: ℞. Ol Ricini; Tr. Opii aa ℥j; Syr. Rhei Av. ℥ij; Gum Arabic ʒij. M. Give a tea spoonful after each evacuation, or once in four hours. To this preparation might be added any pleasant carminatives to conceal the taste and render it less disagreeable. If at the end of twenty-four or forty-eight hours a decided change had not taken place in the discharges, being freer and bilious, I gave: ℞. Mass Hydrarg. ℈j; Pulv. Opii ℈ss. M. Make ten Pills. Give one every 12 hours. Very soon the discharges changed, tenderness and tormina ceased, and the patient recovered. Recoveries in this, as most diseases, were slow. The tongue cleaned in flaky patches, and many had the peculiar shaved appearance.

I have charge of the flag hospital, which is made up of two wards or rooms, separated by a simple board partition, and there is one curious fact in this connection which is inexplicable to me, and that is that the mortality in the two wards is as four to one. The number of patients in the two wards differ but little, and the care and attention on the part of the nurses is the same, and I am very sure there is no want of care in any other way as to ventilation, and still four die in ward eight for every one in ward nine. Neither is there any selection of patients; they are sent indiscriminately to either ward wherever a vacancy occurs. The ventilation of the two

* Our correspondent has incidentally found what is about always true, that Tart. Antim. and Morph. are immensely superior to *Verat. Virid et alii*, in the treatment of Pneumonia.—Ed.

are alike. There is only one fact that seems to bear on it, and that is, that south of ward eight there were a number of privy sinks, and as our principal winds were from the southwest the stench from these came directly through this ward. Whether this was sufficient to account for it, with the free circulation of air which was maintained, or whether it was simply a coincidence, I am unable to determine; but sure I am that it was to me a very curious fact.

This difference was not only for a week, by an accidental accumulation of bad cases, but was maintained during my entire service. Many who recovered of slight ailments, remaining as a matter of comfort or convenience for ration day, relapsed and died. So that at last I allowed none to stay longer than when they were able to go to their quarters.

But to-day the last of our rebel prisoners leave for Vicksburg, and I am detailed to accompany our escort of troops; so that should I not take a conic section into my head, you may hear from me again in Dixie. R**

